# Antifungal Activity of Water-Based Adhesives Derived from Pineapple Stem Flour with Apple Cider Vinegar as an Additive

**DOI:** 10.3390/polym15071735

**Published:** 2023-03-31

**Authors:** Kamonlak Ninsuwan, Jaturavit Nimnuan, Jidapa Watcharakitti, Chomsri Siriwong, Taweechai Amornsakchai, Siwaporn Meejoo Smith

**Affiliations:** 1 Center of Sustainable Energy and Green Materials and Department of Chemistry, Faculty of Science, Mahidol University, 999 Phuttamonthon Sai 4 Road, Salaya 73170, Thailand; 2 Materials Chemistry Research Center, Department of Chemistry and Center of Excellence for Innovation in Chemistry, Faculty of Science, Khon Kaen University, 123 Moo 16, Mittraphap Rd., Nai-Muang, Muang District, Khon Kaen 40002, Thailand

**Keywords:** pineapple stem flour, antifungal agent, apple cider vinegar, transesterification

## Abstract

As a byproduct of bromelain extraction procedures, pineapple stem flour is underutilized. Since water glues derived from gelatinization typically have poor mold resistance, this study aims to produce flour-based value-added products, such as mold-resistant water-based adhesives. To address this issue, this study explored the use of apple cider vinegar (ACV) as a low-cost, non-toxic, commercially available antifungal agent to improve the mold resistance of adhesives. Furthermore, laurate flour was produced via a transesterification of the flour and methyl laurate using a K_2_CO_3_ catalyst. Both the unmodified flour and the functionalized flour were employed to prepare water-based adhesives. For both flour systems, adding ACV at concentrations of at least 2.0% *v*/*v* enhanced the mold resistance of the adhesives and completely inhibited the development of *A. niger* mycelia for up to 90 days of storage. The adhesives made from the transesterified flour exhibited a higher shear strength for the paper bonding (ca. 8%) than the unmodified ones. Additionally, the ACV additive had no negative effects on the shear strengths of the water-based adhesives. All of the flour-based adhesives developed in this study had a higher shear strength for paper substrates than two locally available commercial water glues.

## 1. Introduction

The global adhesives and sealants market is projected to reach approximately USD 85.8 billion by 2026 [1] due to its rapid expansion. Many industries, including the packaging, wood furniture, electronics, and aerospace industries, utilize adhesives as raw materials. In general, formaldehyde, urea, and polyurethanes are the primary constituents of numerous widely used adhesives. However, they are derived from an oil resource. Petroleum, polymers, and adhesives all contain hazardous chemicals. In response to the depletion of fossil fuels, organic solvents, and solvent-based substances, researchers have proposed using biobased alternatives or biodegradable “green materials” to replace petrochemicals in adhesive components [2,3]. Due to its low cost, renewable nature, and widespread availability, starch has an advantage as a potentially renewable resource with a low price in the adhesive industry. Native starches, which are polysaccharides composed of amylose and amylopectin, are generally hydrophilic due to the presence of hydroxyl groups in their structures [4,5]. Consequently, adhesives derived from native starches are less prevalent on the market as they have low water resistance [4,5,6], resulting in poor adhesive performance. Therefore, researchers have been focusing on modifying the structures of native starches to improve their adhesive properties and water resistance [7]. In addition, water-based adhesives from starch have a problem with shelf life due to microbial attacks. *Aspergillus niger*, a black mold disease, usually contaminates food, especially sun-dried foods, grains, and nuts. Some strains of this fungus can also produce the mycotoxin ochratoxin A, which is responsible for human kidney damage. Additionally, the molds can also attack low-water-resistance adhesives. This is because many low-water-resistance adhesives can easily be penetrated by moisture, and the molds take advantage of this to grow on the surfaces of the adhesives [8,9,10]. Jin et al. reported an antibacterial adhesive derived from silicone, catecholamine-based tannic acid, and copper ions, which create a synergistic antimicrobial effect against *E. coli* and *S. aureus* [11]. Combining polymer, urea with carvacrol oil and mixing them to form supramolecular adhesives inhibited the growth of Gram-positive and Gram-negative bacteria for up to 70 days [12]. In addition, the essential oils *Mentha pulegium* and *Lavandula angustifolia* were mentioned to inhibit the growth of *S. aureus* in starch films [13]. Additionally, lemongrass oils (*Cymbopogon citratus*), neem oil, and oregano oils in starch-based films showed antifungal activity against *Aspergillus niger* (*A. niger*) [14,15].

Vinegar is used in the food industry as a preservative by inhibiting fungal growth on vegetables due to its high content of organic acids, phenolic compounds, tannins, flavonoids, and carotenoids with antimicrobial activity [16,17,18]. These properties make vinegar an effective preservative that increases the shelf life of foods and prevents spoilage. Due to its medicinal properties, it is also used as a cleaning agent in folk culture to treat wounds by inhibiting the growth of fungi and bacteria. Vinegar is a highly versatile substance with a wide range of applications, ranging from use as a natural preservative method for inhibiting the growth of foodborne pathogenic microorganisms in food to medicinal or point-of-care uses, such as nail care and treatment, lowering bad cholesterol, having anti-diabetic and anti-Alzheimer properties [19,20,21], and household uses, including trapping fruit flies, cleaning and deodorizing appliances, and solving clogged drain problems [22].

This study aimed to assess apple cider vinegar’s in vitro antifungal properties on water-based adhesives made from pineapple stem flour because of its simplicity of use, abundance, low cost, and low toxicity. In order to test the viability of using apple cider vinegar as an antifungal reagent in polysaccharide-based adhesives rather than harmful synthetic chemicals, the shear strength of starch adhesives was measured and compared with that of other commercially available paper glues. The key findings from this work should promote the value creation of starch utilizations as natural raw materials for the development of biobased water glues for paper.

## 2. Materials and Methods

Hong Mao Biochemicals Co., Ltd. (Rayong, Thailand) supplied the pineapple stem flour (PAF), which is a by-product of bromelain extractions (Rayong, Thailand). Before further use, the flour was dried at 65 °C for 48 h. The commercially available analytical-grade chemicals that were used include dimethyl sulfoxide (DMSO; Scharlab S.L., Barcelona, Spain), potassium carbonate (K_2_CO_3_; MERCK, Darmstadt, Germany), methyl laurate (Tokyo Chemical Industry, TCI, Tokyo, Japan), and ethanol (AR grade; RCI Labscan Limited, Bangkok, Thailand). Water (RO grade; conductivity = 5–15 microsiemens) was used to prepare the water glue samples. Apple cider vinegar (5 wt.%; pH 2.21; ECOVINAL S.L., Sartaguda, Navarra, Spain) was purchased from a local supermarket in Nakorn Pathom, Thailand.

Potato dextrose agar (HIMEDIA, Maharashtra, India) and potato dextrose broth (HIMEDIA, Maharashtra, India) were also used for this study. *Aspergillus niger* TISTR3281 (or *A. niger*) was purchased from the TISTR Biodiversity Research Center, Pathum Thani, Thailand. Cardboard paper (100 g) was supplied by PT TRANG CO., LTD., Trang, Thailand. Two commercial water glues were purchased from local shops in Nakorn Pathom, Thailand.

### 2.1. Modified PAF (m-PAF)

In a 500 mL round-bottom flask equipped with a magnetic stir bar, a suspension of the PAF flour (5 g) in 50 mL of DMSO was kept at 75 °C for 3 h to allow for effective starch gelatinization processes. Subsequently, K_2_CO_3_ (0.25 g) and methyl laurate (19.81 g) were added to the gelatinized mixture. The transesterification reaction between the PAF flour and methyl laurate underwent at 110 °C for 1 h. The reaction was subsequently cooled to room temperature. The resultant solid product was suspended in 200 mL of ethanol, agitated for 15 h, and then washed three times with ethanol. After filtering and drying in an air oven at 65 °C for 15 h, the esterified starch product (denoted as m-PAF) was obtained as a light-yellow powder. The molecular weight of the PAF and m-PAF samples were determined by gel permeation chromatography using Agilent Waters 2414, column: TSKgel G5000PW (300 × 7.5), in water as the solvent. The transesterification efficiency was measured in terms of the degree of substitution (DS) of the ester group on the starch backbone using a 1H NMR spectrometer (Bruker AVANCE 600 series FT NMR spectrometer (600 MHz)). The chemical shifts were reported in ppm (d6–DMSO at 2.50 ppm). The degree of substitution was determined as follows: 10 mg of m-PAF was added into a 1.5 mL screw-cap vial equipped with a magnetic stir bar. Around 1000 µL of deuterate solvent as d6–DMSO was added and stirred at 60 °C for 3 h. After that, the 700 µL mixture was transferred to an NMR tube. The signal was recorded as follows: 32 scans at 50 °C. The degree of substitution (DS) was determined by Equation (1) [23], which is as follows:

(1) $$\mathrm{DS}\;=\;\frac{I_{\left(0.85\;\mathrm{ppm}\right)}/3}{I_{\left(3.6–5.4\;\mathrm{ppm}\right)}/7}$$

where *I* (0.85 ppm) refers to the integration of the proton signal corresponding to the terminal position of the laurate chain at C-12 and *I* (3.6–5.4 ppm) refers to the integration of the proton signal of the glucose unit.

### 2.2. Water-Based Adhesives

One gram of either PAF or m-PAF and 20 mL of water were placed in a clear zipper polypropylene bag. The mixture was then manually agitated in the zipper bag, before being heated seven times in a household microwave oven (800 watts; MS23F300EEK; SAMSUNG, Kuala Lumpur, Malaysia) for one minute at a time. Then, apple cider vinegar (ACV) was added into the aforementioned flour mixture in proportions of 0.3, 0.625, 1.25, 2, 2.5, and 3% *v*/*v*. A light-yellow water glue was obtained.

### 2.3. Antifungal Activity Test

*A. niger* is a representative of the fungi family and is easily found in starchy foods. The fungal strain was incubated in a potato dextrose broth (PDB) at 35 °C for 7 days. The suspension formed by *A. niger* and spores was collected and transferred to a sterile tube. The optical density (OD) of the suspension was adjusted to 0.05 using a UV–visible spectrophotometer at 600 nm, adapted by Su et al. and Larregle et al. [24,25].

A mixture containing 39 g of mixed PDA powder in 1 L of RO water was sterilized in an autoclave (LS50HD; LAUSON, Hangzhou West Tune Trading Co., LTD., Hangzhou, China) at 121 °C for 15 min. The previously prepared water glue samples were then used to monitor the mycelial development of the *A. niger* fungus. First, 5 mL of the adhesive was mixed with 25 mL of the prepared aqueous PDA solution in a sterile tube, after which the mixture was placed in a sterile petri dish (13 mm in diameter). Sterile disks with a diameter 6 mm were soaked in the suspension of *A. niger*. To evaluate the antifungal activity of the ACV-containing water glues, the fungus-containing sterile disks were placed at the center of the agar medium mixed with the glue at varying ACV concentrations. The petri dishes were incubated at room temperature for up to 90 days. The fungal mycelial growth was monitored. The effect of the ACV concentrations in the PAF (or m-PAF) water-based adhesives on the fungal mycelial growth was calculated in terms of the percentage of mycelial growth inhibition (MGI) using the following Equation (2) [26]:

(2) $$\mathrm{MGI}\;(\%)=\frac{dc-dt}{dc}\times 100$$

where *d*c = the mean diameter (mm) of the fungal mycelial growth in the control treatment and *d*t = the mean diameter (mm) of the fungal mycelial growth in the sample-containing treatment.

### 2.4. Shear Strength Test

**Figure 1:**
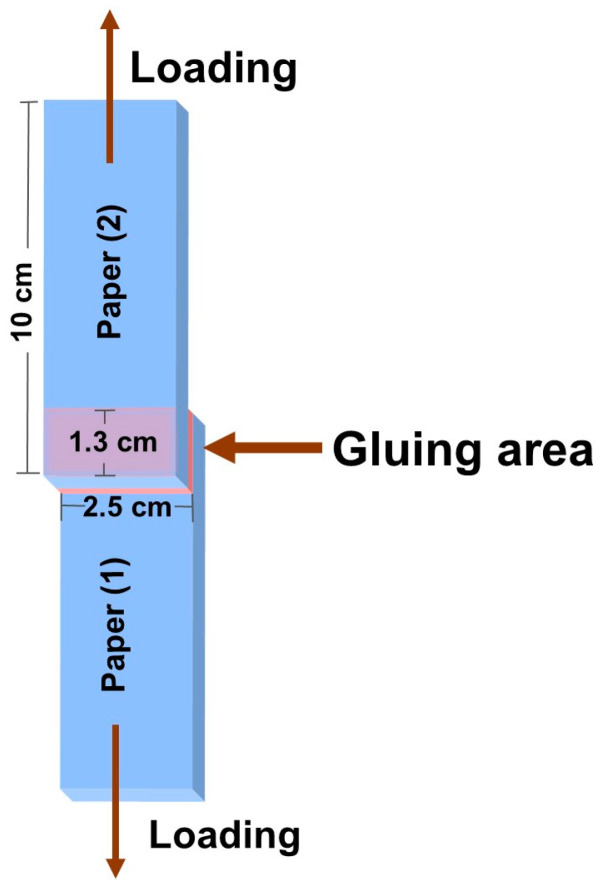
Schematic of shear strength on the paper substrate.

For each test, two pieces of paperboard were cut to a width of 2.5 cm and a length of 10 cm. The adhesive application area (2.5 cm × 1.3 cm) was positioned at the end of each piece (see Figure 1). A painting brush (No. 7) was used to apply 40 g of each water-based glue to the gluing area, where the paperboard widths were overlapped. The paperboard adhesive was then allowed to dry at room temperature. A universal testing machine was used to measure the adhesive’s shear strength (INSTRON 5965 Norwood, MA, USA; 5 kN max capacity), followed by a modification of the ASTM D1002 method with a tension mode and a crosshead speed of 5 mm/min [27].

Shear strength (MPa) = Maximum loading force (N)/Bonding area (m^2^) (3)

### 2.5. Statistical Analysis

The results were reported as a mean value and standard deviation (SD). The descriptive statistics were calculated using version 18 of the predictive analytics software (PASW) program [28]. A one-way analysis of variance (ANOVA) was used to test the growth of the fungi at each ACV concentration, followed by a Duncan’s multiple range test to determine significance (*p*-values ≤ 0.05).

## 3. Results

Based on the GPC and 1H NMR results, the properties of PAF and m-PAF are shown in Table 1.

The molecular weight of PAF (106 Da) lies in the same range as other starches, including wheat starches (6.5 × 10^6^ Da) [29], 51% of amylose corn-based starches (6.2 × 10^6^ Da) [30], and 80% of amylose corn-based starches (1.9 × 10^6^ Da) [31]. The polydispersity indices of PAF and m-PAF are similar and close to 1.00, demonstrating that m-PAF has the same molecule distribution properties as PAF. The maximum value of the degree of substitution (DS) is 3.00; therefore, a value of 0.13 indicates moderate ester group functionalization on the starch backbone.

### 3.1. ACV/PAF Water-Based Adhesives

#### 3.1.1. Antifungal Property

**Figure 2:**
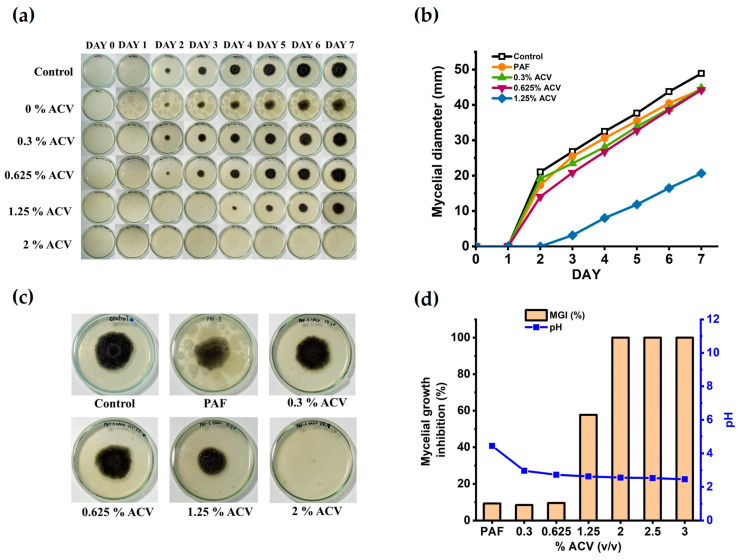
The mycelial growth of *A. niger* on the adhesive on the agar medium was effectively inhibited by the addition of ACV at the optimum concentrations. (**a**) Seven-day monitoring for any microorganism growth in the PAF and ACV/PAF water glues spiked with the *A. niger* fungi; (**b**) the measured mycelial diameter (mm) of the fungal growth; (**c**) the fungal growth after the seven-day storage under air exposure; (**d**) the percent of mycelial growth inhibition at various ACV concentrations in the adhesives and their pH.

Figure 2a shows the time-dependent mycelial growth of *A. niger* in the water glues derived from the unmodified starch (PAF) and ACV/PAF mixtures under controlled conditions (room temperature). With moisture in the air, mold and bacteria easily attacked the adhesives produced from the unmodified starch. The round black shades in the figures reflect the growth of the fungi in the adhesive, while the white spots represent some bacterial colonies (see also Figure S1 in the Supplementary Information). Nevertheless, adding a small amount of ACV slowed mold formation, as evidenced by the staining of the samples on the fourth day for the PAF adhesive containing 0.125% *v*/*v* ACV. The mold and bacterial resistance of the ACV/PAF mixture was substantially enhanced by increasing the concentration of the ACV to 2% *v*/*v*. Neither black nor white stains were observed in the PAF adhesives with 2% *v*/*v* ACV and above.

Figure 2b displays the quantitative *A. niger* mycelial growth data as the growth diameters. According to the statistical analysis results in Table 2, an ACV addition range of 0–1.25% *v*/*v* was insufficient to promote the growth of microorganisms in the ACV/PAF water-based adhesives (*p* ≤ 0.05). Although smaller black shades of mold growth were observed with the increased ACV concentrations, as seen in Figure 2c, complete mycelial growth inhibition requires a concentration of ACV in the adhesive solutions of at least 2.0% *v*/*v*. Figure 2d also confirms that the mycelial growth inhibition reached 100% for the ACV/PAF adhesive with an ACV concentration of 2.0% *v*/*v* and above. In line with a previous report [32], adding ACV to the adhesive solutions made them more acidic (pH < 2.6 at an ACV concentration of ≥ 2.0% *v*/*v*). It is possible that the improved antifungal activity of the ACV/PAF mixtures was due to the lower cellular pH (from 4.5 to 2.6). According to Kara et al., acidity is associated with antimicrobial activity. The antibacterial activity against *S. aureus* and *E. coli* (ATB: 57) was enhanced by the low pH of apple vinegars (RD and Gala) [33].

**Figure 3:**
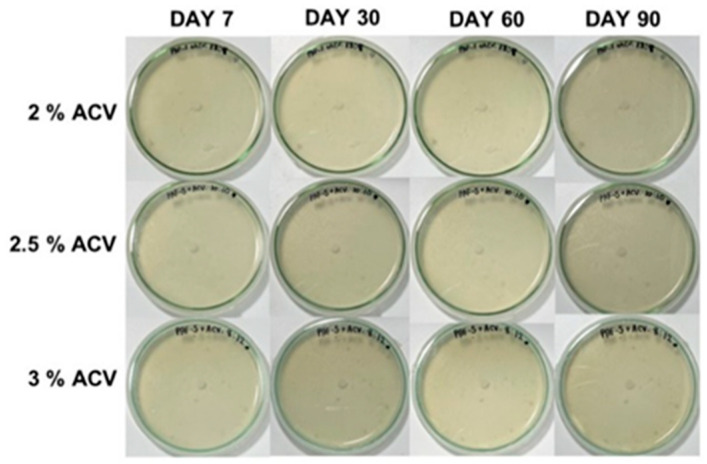
The fungal growth in the adhesives derived from the PAF or ACV/PAF mixtures at various ACV concentrations after 7, 30, 60, and 90 days of storage.

Due to the high mold resistance of the water-based adhesives because of the optimum ACV concentrations, no fungal growth was observed for very long storage of up to 90 days (see also Figure 3). As a result, ACV can be used as an effective antifungal agent in flour-based adhesives. As shown in Figure S1 in the Supplementary Information, the mycelial growth of the *A. niger* fungus can be seen as dark shades on the plate, and the white spots in the PAF and m-PAF samples are showing evidence of some bacterial growth. When 0.3% ACV is combined with the adhesives, the white spots disappear, implying inhibited bacterial growth. This result agrees well with previous work reported by Baysal et al. [18]. Due to its high phenolic content, ACV has been shown to have antibacterial activity against both Gram-positive (*L. monocytogenes* and *S. aureus*) and Gram-negative (*E. coli* and *Salmonella*) bacteria [18].

#### 3.1.2. Shear Strength of the ACV/PAF Adhesives

**Figure 4:**
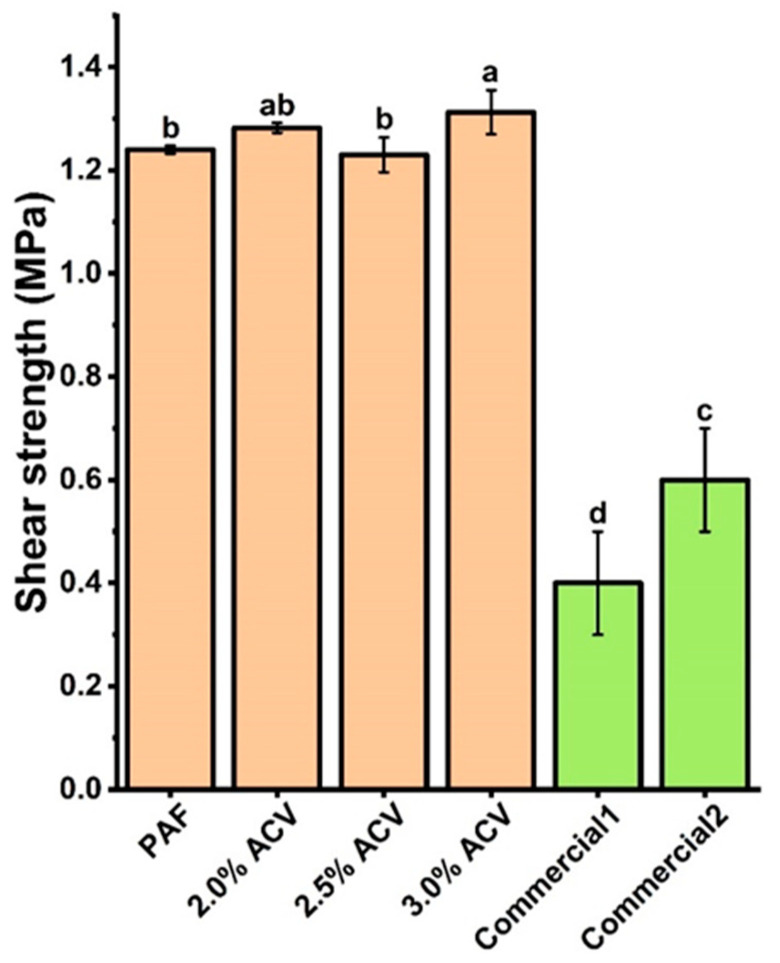
The shear strengths of the PAF and ACV/PAF adhesives on the paper substrates compared with two commercially available water glues. Letter notation on top of the bar-graphs indicated group classified by statistical analysis. Similar notation means that data are statistically the same (*p* ≤ 0.05).

The ACV/PAF adhesive with 3.0% *v*/*v* ACV shows the highest shear strength for the paper substrate (Figure 4), while the shear strengths obtained from the ACV/PAF adhesives at 2.0 and 2.5% *v*/*v* ACV are not significantly different (*p* ≤ 0.05). Nevertheless, the high shear strength values of 1.25–1.35 MPa for all adhesives studied suggested that the addition of ACV does not deteriorate the mechanical properties of the flour-based adhesive. Notably, all of the produced adhesives have significantly superior shear strengths to two commercially available water glues.

### 3.2. ACV/m-PAF Water-Based Adhesives

#### 3.2.1. Antifungal Activity Test

**Figure 5:**
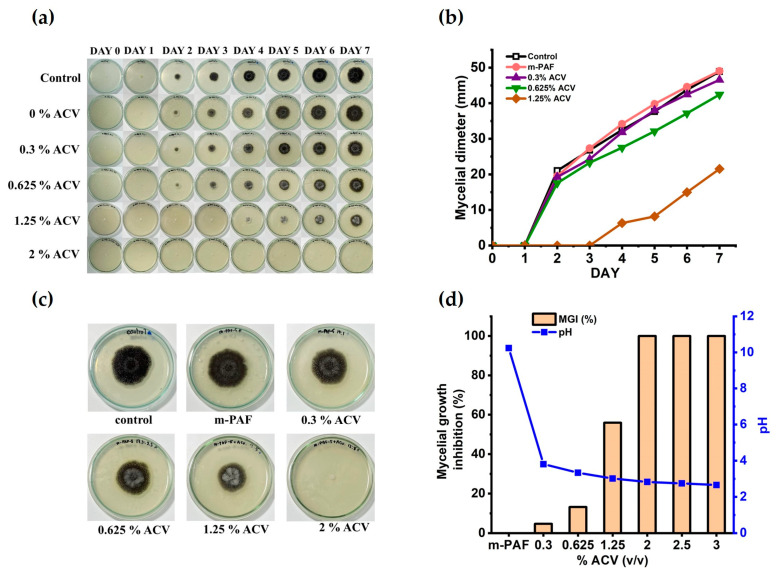
The mycelial growth of *A. niger* on the m-PAF and ACV/m-PAF adhesives in agar media was monitored for seven days. (**a**) Photos of the mycelial growth during the seven days of storage; (**b**) the diameter (mm) of fungal growth on days 0–7; (**c**) the mycelial growth after seven days of storage; (**d**) mycelial growth inhibition (%) at varying ACV concentrations.

On the second day of storage, as shown in Figure 5a, the m-PAF adhesive begins to develop black patches. This indicates that the esterified PAF, by itself, is not mold-resistant. Comparing the diameters of mycelial development in Figure 2a,b and Figure 5a,b, ester functionalization marginally improves the antifungal activity of the flour-based adhesives. Similar antifungal activity of the ACV/m-PAF adhesives with an ACV concentration of 0.625% *v*/*v* and below was observed based on the results in Figure 5a,b and Table 3 (not significantly different; *p* ≤ 0.05). At an ACV concentration of 1.25% *v*/*v*, the mycelial growth inhibition improved moderately. In the examined ACV/m-PAF water-based adhesive, the 2.0% *v*/*v* ACV concentration effectively inhibits the growth of fungi and bacteria (Figure 5c and Figure S1). Notably, the white spots, which were due to bacterial growth, were observed on the ACV/m-PAF adhesives with an ACV concentration of 0%, similar to the ACV/PAF case (Figure 2a). In the same way, adding 2.0% *v*/*v* ACV to the ACV/m-PAF adhesive made it resistant to mold and stopped the fungal growth (Figure 5c).

**Figure 6:**
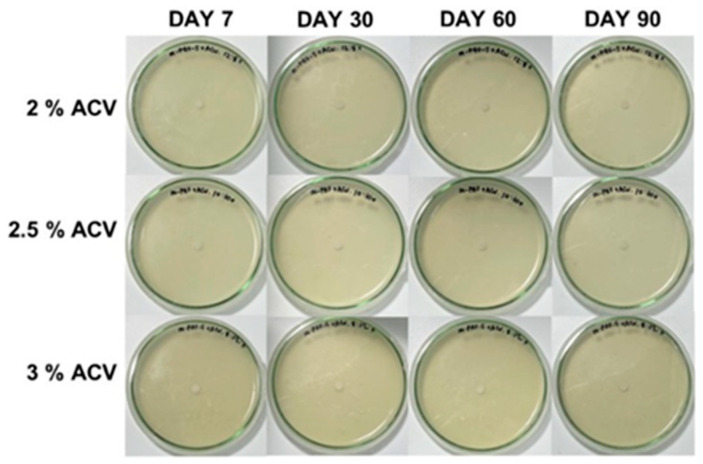
The growth of *A. niger* in the m-PAF and ACV/m-PAF adhesives after 7, 30, 60, and 90 days of storage.

As shown in Figure 5d, the m-PAF has a highly basic pH of 10.2, possibly due to some residual base catalyst (K_2_CO_3_) in the m-PAF. By adding a small amount of ACV, the pH of the ACV/m-PAF adhesives is found to be much lower than that of the m-PAF one. The gradual decrease in pH of the ACV/m-PAF adhesives due to the increasing ACV concentrations occurred along with a significant improvement in the antifungal activity of the ACV/m-PAF adhesives. With an ACV concentration of 2.0% *v*/*v* or more, the ACV/m-PAF adhesives could completely stop the fungal and bacterial growths (as shown in Figure S1). This antifungal activity of ACV makes the adhesives resistant to mold for up to 90 days (Figure 6).

#### 3.2.2. Shear Strength of ACV/m-PAF Water-Based Adhesives

**Figure 7:**
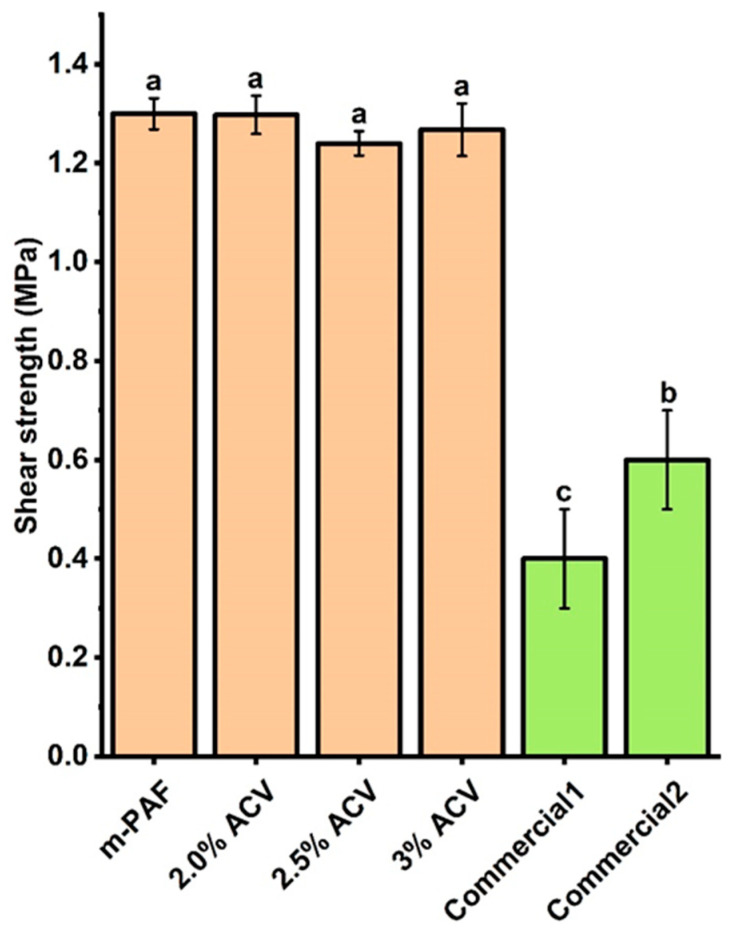
Shear strengths of the m-PAF and ACV/m-PAF adhesives at various ACV concentrations. Group classification (a, b, and c) was given to the data based on the statistical analysis results (significant difference; *p* ≤ 0.05). Green bar graphs are given for the results from commercially available water glues.

In comparison, the shear strengths of the papers obtained from the m-PAF-based adhesives are slightly higher (ca. 8%) than those of the PAF water glues (Figure 4 and Figure 7). Notably, two commercially available water glues showed much lower shear strengths compared to the produced flour-based adhesives. From the results in Figure 7, adding more ACV to the ACV/m-PAF adhesives does not modify the mechanical properties of the adhesives. Similar shear strengths (insignificantly different; *p* ≤ 0.05) were obtained for the ACV/m-PAF water-based adhesives at ACV concentrations of 2.0–3.0% *v*/*v*. Thus, the results suggest the feasibility of utilizing ACV as an effective antifungal agent in water glues, as it can extend the glues’ shelf life without worsening their mechanical properties.

**Figure 8:**
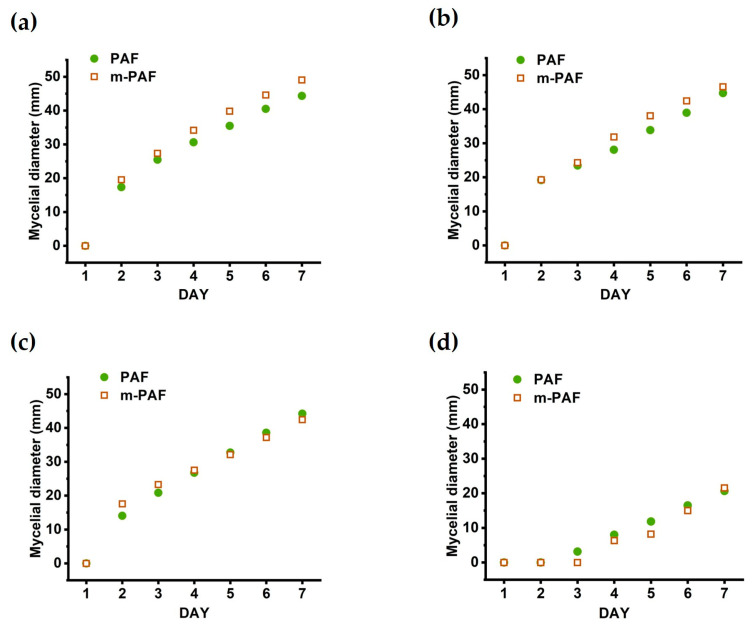
Comparative growth of *A. niger* in PAF and m-PAF mixed with ACV (**a**) 0% *v*/*v*; (**b**) 0.3% *v*/*v*; (**c**) 0.625% *v*/*v*; (**d**) 1.25% *v*/*v*.

To better understand the effect of ACV in suppressing mycelial growth in the investigated adhesives, the mycelial diameters in the PAF and m-PAF systems were compared in Figure 8. Notably, the ACV has a 1% wt of sugar that can enhance fungal growth in the flour adhesives at low ACV concentrations. Although both systems required a minimum ACV concentration of 2.0% *v*/*v* to produce mold-resistant flour-based adhesives, it is important to note that the mycelial growth rate of these adhesives differs at ACV concentrations that are less than 2.0% *v*/*v*. Despite the small improvement in the mechanical properties of the esterified flour adhesive compared to the adhesive produced from the unmodified flour, it is not entirely understood whether the ester groups functionalized on the polysaccharide backbone play an important role in the antifungal activity of flour-based adhesives. From Figure 8a, the faster mycelial growth in m-PAF is possibly due to the basic pH of the m-PAF adhesive creating a more suitable medium for fungal growth. Similarly, at a low ACV concentration of 0.3% *v*/*v*, the high pH of the gelatinized ACV/m-PAF may be the major cause of the somewhat quicker mycelial development and lower mold resistance in the ACV/m-PAF. On the other hand, at increased ACV concentrations, the mycelial development rates were found to be quite similar for both the ACV/PAF and ACV/m-PAF cases. Hence, adding a suitable amount of ACV to flour-based adhesives could be more effective in enhancing antifungal activity than using modified flour as an adhesive raw material. As previously reported [34], pH plays an important role in the growth rate of fungi, and a pH of around 5.5–7.0 is most suitable for fast fungal growth. The faster growth of *A. flavus* at aw 0.98 and pH 5.5 compared to pH 3.0 [35] and the fast development of *A. niger* mycelia in the medium having a pH of around 7.0 [36] support the idea that pH ≥ 5.5 is suitable for boosting the mold development rates. In the PAF adhesives, the acetic acid, organic acids (maleic acid, lactic acid, and tartaric acid [37]), and polyphenolic compounds in the ACV may act as antifungal agents [38]. The acids lowered the pH of the flour-based adhesives and the cellular pH of the fungi [39]. Diffusion of the acids occurred across the plasma membrane of the fungal cells. Thus, inhibiting nutrient transport led to the protonation of cell macromolecules, destabilizing the fungi and inducing their death [40,41,42,43]. Moreover, ACV impairs cell integrity, organelles, and protein expression [20]. The phenolic compounds in fruit-based vinegars could inhibit the fungal growth processes and disturb the integrity of membrane cells [38]. The result of this work agreed well with another previous study [34], such that sorbic acid (with a similar pKa to acetic acid) effectively delayed spore germination and decreased the biomass yields of *A. niger*. This growth inhibition was concentration-dependent, with the greater acid concentrations having the greatest impact.

In the m-PAF adhesives, the addition of ACV caused the formation of salts (acetate, maleate, lactate, and tartrate salts) due to acid–base neutralization between the basic gelatinized m-PAF solution (pH 10.2) and the acids in the ACV. Salinity effects have been reported to inhibit fungal mycelial development [44,45]. The salts disrupt the metabolic activities in fungal cells, and they may interfere with the osmotic equilibrium of the fungal cells as a result of the “salting out” action, resulting in the coagulation of the protein components of fungal cells [46]. In addition, several researchers have shown that acetate can block the enzyme lanosterol 14α-demethylase by lowering ergosterol synthesis, which influences membranes and, thereby, disrupts fungal development. Acetate decreases the enzyme activity in ergosterol production, causing membrane damage [37]. Due to the salt formation in the ACP/m-PAF systems at ACV concentrations of 0.625% and 1.25% *v*/*v*, the salts in the modified flour-based adhesives inhibit fungal growth more effectively than the salt-free adhesives produced from PAF. Previous works have demonstrated the antifungal properties of ACV; however, this is the first study to investigate the use of ACV in adhesive applications. Table 4 summarizes the antifungal activity promoted by ACV and fruit vinegars in previous research and this study.

## 4. Conclusions

This study has demonstrated that apple cider vinegar (ACV) has great potential as an antifungal agent, significantly enhancing the fungal resistance of water-based adhesives made from pineapple stem flour (PAF) and esterified PAF (modified PAF or m-PAF). By adding ACV at a low concentration of 2.0% *v*/*v* to flour-based adhesives, it is possible to obtain long-shelf-life water glues with high antifungal activity against *A. niger*, a common fungus found in starchy foods. The chemical composition (organic acids and polyphenols) and pH of ACV should play an important role in the satisfactory antifungal activity of the ACV-containing adhesives. Additionally, the bonding strength of the adhesives on the papers suggested that ester functionalization slightly improved the mechanical properties of the flour adhesives. The results in this work indicated that pineapple stem flour, a low-cost, abundant, and underutilized source of polysaccharides, is applicable as a biobased raw material for the production of eco-friendly water-based adhesives that give high bonding strengths to paper substrates and are superior to two commercially available water glues. The key findings of this study support the viability of producing biobased adhesives with a cheap, non-toxic, and naturally based antifungal agent, which serves well from the perspective of health-conscious customers. Therefore, the novelty of this work should serve as a useful resource for further research and development of additional low-cost, eco-friendly, biobased products in adhesive and other applications.

## Figures and Tables

**Table 1:** Properties of the pineapple stem flour (PAF) and modified pineapple stem flour (m-PAF).

Sample	M_w_ (Da)	M_n_ (Da)	Polydispersity Index	Degree of Substitution
PAF	2.02 × 10^6^	1.97 × 10^6^	1.03	0.00
m-PAF	3.44 × 10^6^	3.32 × 10^6^	1.03	0.13

**Table 2:** Mycelial diameter (mm) of the *A. niger* fungi in the PAF and ACV/PAF adhesives. The results are reported as mean ± SD, while the group classifications (a, b, c, and d) within each column are listed based on the statistical analysis (significant differences; *p* ≤ 0.05) by using a Duncan’s multiple range test. The statistical analysis was conducted on each of the data columns.

Sample/Day	Mycelial Growth (mm)							
	0	1	2	3	4	5	6	7
Control	0.0 ^a^	0.0 ^a^	21.0 ± 1.5 ^a^	26.8 ± 1.5 ^a^	32.5 ± 1.7 ^a^	37.7 ± 1.4 ^a^	44.6 ± 1.1 ^a^	48.9 ± 0.6 ^a^
PAF	0.0 ^a^	0.0 ^a^	17.3 ± 0.8 ^b^	25.5 ± 0.8 ^a^	30.6 ± 1.0 ^a^	35.5 ± 1.0 ^a^	42.5 ± 1.3 ^a^	44.4 ± 0.8 ^a^
0.3% ACV/PAF	0.0 ^a^	0.0 ^a^	19.2 ± 0.2 a^b^	23.5 ± 0.6 ^ab^	28.1 ± 0.6 ^a^	33.9 ± 1.8 ^a^	38.9 ± 2.0 ^a^	44.7 ± 1.1 ^a^
0.625% ACV/PAF	0.0 ^a^	0.0 ^a^	14.1 ± 2.9 ^c^	20.9 ± 1.5 ^b^	26.8 ± 1.9 ^a^	32.8 ± 0.7 ^a^	38.6 ± 1.2 ^a^	44.2 ± 1.7 ^a^
1.25% ACV/PAF	0.0 ^a^	0.0 ^a^	0.0 ^d^	3.2 ± 5.5 ^c^	8.0 ± 7.7 ^b^	11.9 ± 11.4 ^b^	16.5 ± 15.1 ^b^	20.7 ± 18.8 ^b^
2.0% ACV/PAF	0.0 ^a^	0.0 ^a^	0.0 ^d^	0.0 ^c^	0.0 ^c^	0.0 ^c^	0.0 ^c^	0.0 ^c^
2.5% ACV/PAF	0.0 ^a^	0.0 ^a^	0.0 ^d^	0.0 ^c^	0.0 ^c^	0.0 ^c^	0.0 ^c^	0.0 ^c^
3.0% ACV/PAF	0.0 ^a^	0.0 ^a^	0.0 ^d^	0.0 ^c^	0.0 ^c^	0.0 ^c^	0.0 ^c^	0.0 ^c^

**Table 3:** The mycelial diameter (mm) of *A. niger* in the m-PAF and ACV/m-PAF adhesives at varying concentrations of ACV within seven days of storage. The results are reported as mean ± SD, while the group classifications (a, b, c, and d) within each column are listed based on the statistical analysis (significant differences; *p* ≤ 0.05) by using a Duncan’s multiple range test. The statistical analysis was conducted on each of the data columns.

Sample/Day	Mycelial Growth (mm)							
	0	1	2	3	4	5	6	7
Control	0.0 ^a^	0.0 ^a^	21.0 ± 1.5 ^a^	26.8 ± 1.5 ^a^	32.5 ± 1.7 ^a^	37.7 ± 1.4 ^a^	44.6 ± 1.1 ^a^	48.9 ± 0.6 ^a^
m-PAF	0.0 ^a^	0.0 ^a^	19.5 ± 0.8 ^b^	27.3 ± 1.5 ^a^	34.2 ± 0.8 ^a^	39.8 ± 0.9 ^a^	44.6 ± 1.6 ^a^	49.1 ± 2.7 ^a^
0.3% ACV	0.0 ^a^	0.0 ^a^	19.3 ± 1.1 ^b^	24.3 ± 1.8 ^b^	31.9 ± 4.5 ^a^	38.0 ± 5.3 ^a^	42.5 ± 5.3 ^a^	46.6 ± 3.1 ^a^
0.625% ACV	0.0 ^a^	0.0 ^a^	17.6 ± 1.0 ^c^	23.3 ± 0.4 ^b^	27.5 ± 0.8 ^a^	32.1 ± 1.2 ^a^	37.2 ± 0.8 ^a^	42.4 ± 0.5 ^a^
1.25% ACV	0.0 ^a^	0.0 ^a^	0.0 ^d^	0.0 ^c^	6.31 ± 1.0 ^b^	8.2 ± 14.2 ^b^	15.0 ± 14.2 ^b^	21.5 ± 13.8 ^b^
2.0% ACV	0.0 ^a^	0.0 ^a^	0.0 ^d^	0.0 ^c^	0.0 ^b^	0.0 ^b^	0.0 ^c^	0.0 ^c^
2.5% ACV	0.0 ^a^	0.0 ^a^	0.0 ^d^	0.0 ^c^	0.0 ^b^	0.0 ^b^	0.0 ^c^	0.0 ^c^
3.0% ACV	0.0 ^a^	0.0 ^a^	0.0 ^d^	0.0 ^c^	0.0 ^b^	0.0 ^b^	0.0 ^c^	0.0 ^c^

**Table 4:** Reported antifungal activities of apple cider vinegars (ACVs) and vinegars produced from fruits.

Organism	Antifungal Agent	Test Method	Diameter of Zone Inhibition/Inhibition Efficiency	Remark	Ref.
*Candida albicans*	ACV	Agar well diffusion method	12 mm	Cell integrity damage, Disturbed structure of metabolic protein, and nuclei.	[20]
*Candida albicans* (ATCC 18804)	ACV and fruit vinegar	Agar well diffusion method	11 mm	Impaired cell integrity, Disrupted organelles and protein expression.	[47]
	Fruit vinegar		20.5 mm		
*Candida tropicalis*	ACV	Agar well diffusion method	11–12 mm	Acidic pH induced the fungi membrane cell destruction. Phenolics induced growth inhibition of microbes. Polyphenols disturb the membrane cells integrity.	[17]
*Candida albicans* *Aspergillus niger*	ACV	Agar well diffusion method	*C. albicans*; 20.8 ± 0.4 mm	Lowering of cellular pH.	[32]
			*A. niger*; 26.6 ± 0.5 mm		
*Candida albicans*	ACV (1, 10, 25, 50, 100%)	Agar well diffusion method	100% ACV; 6 mm	Phenolic compounds are antifungal agents.	[48]
*Aspergillus niger*			100% ACV; 25 mm		
			50% ACV; 10 mm		
			25% ACV; 3 mm		
*Candida albicans* DSMZ, 1386	ACV	Minimum inhibitory concentration (MIC)	Ineffective	-	[19]
	Grape vinegar		50 ug/mL		
*Escherichia. coli* *Pseudomonas aeruginosa* *Klebsiella pneumonia * *Staphylococcus aureus*	Apple vinegar	Agar well diffusion method	*E. coli*; 17.3 ± 0.6 mm	By inhibiting bacterially produced -lactamases and topoisomerase enzymes, flavonoids can also inactivate the efflux pump and cause the cytoplasmic membrane to become unstable. Catechins cause lipopolysaccharide to degrade, and the degradation products function as a barrier in the bacterial membranes.	[33]
			*P. aeruginosa*; 32.7 ± 2.5 mm		
			*K. pneumonia*; 17.3 ± 0.6 mm		
			*S. aureus*; 20.7 ± 1.2 mm		
		Minimum inhibitory concentration (MIC) (µL/mL)	*E. coli*; 7.81		
			*P. aeruginosa*; 3.91		
			*K. pneumonia*; 3.91		
			*S. aureus*; 7.81		
		Minimum bacterial concentration (MBC) (µL/mL)	*E. coli*; 15.62		
			*P. aeruginosa*; 7.81		
			*K. pneumonia*; 15.62		
			*S. aureus*; 15.62		
*Listeria monocytogenes Staphylococcus aureus * *Escherichia coli * *Salmonella*	Chitosan encapsulated with ACV	Agar well diffusion method	*L. monocytogenes*; 908.0 ± 0.0 mm^2^	Phenolic compounds are antifungal agents.	[18]
			*S. aureus*; 314.0 ± 0.0 mm^2^		
			*E. coli*; 201.0 ± 0.0 mm^2^		
			*Salmonella*; 314.0 ± 0.0 mm^2^		
*Aspergillus niger*	ACV in water glue derived from the native PAF starch	Mycelial growth inhibition	2–3% *v*/*v*, 100% inhibition	Organic acids, phenolics in ACV are antifungal agents.	This study
	ACV in water glue derived from the esterified PAF starch			Antifungal agents are organic acids, phenolics in ACV, and salts generated from acid-base neutralization.	This study

## Data Availability

The data presented in this study are available on request from the corresponding author.

## Supplementary Materials

The following supporting information can be downloaded at: https://www.mdpi.com/article/10.3390/polym15071735/s1, Figure S1: Microorganism growth monitored over a 7-day period in the adhesive systems. (a) control, (b) PAF water glue, (c) PAF with 0.3% ACV water glue, (d) PAF with 2% ACV water glue, (e) m-PAF water glue, (f) m-PAF with 0.3% ACV water glue, and (g) m-PAF with 2% ACV water glue. In unmodified starch (PAF) and starch ester (m-PAF) adhesives, ACV was employed at varying concentration as an antifungal agent against the *A. niger* fungus. The mycelial growth of *A. niger* fungus can be seen as dark shade in the plate, and white spots in PAF and m-PAF sample are the evidence of some bacterial growth. When 0.3% ACV is combined with the adhesives, white spots disappear.
