# Supplementary material for: Antifungal Activity of Water-Based Adhesives Derived from Pineapple Stem Flour with Apple Cider Vinegar as an Additive
Source: Polymers (Basel). 2023 Mar 31;15(7):1735. doi: 10.3390/polym15071735 (PMC10097009; doi:10.3390/polym15071735)
Supplement: Supplementary file 1 [file polymers-15-01735-s001.zip › polymers-2298425-supplementary.pdf]

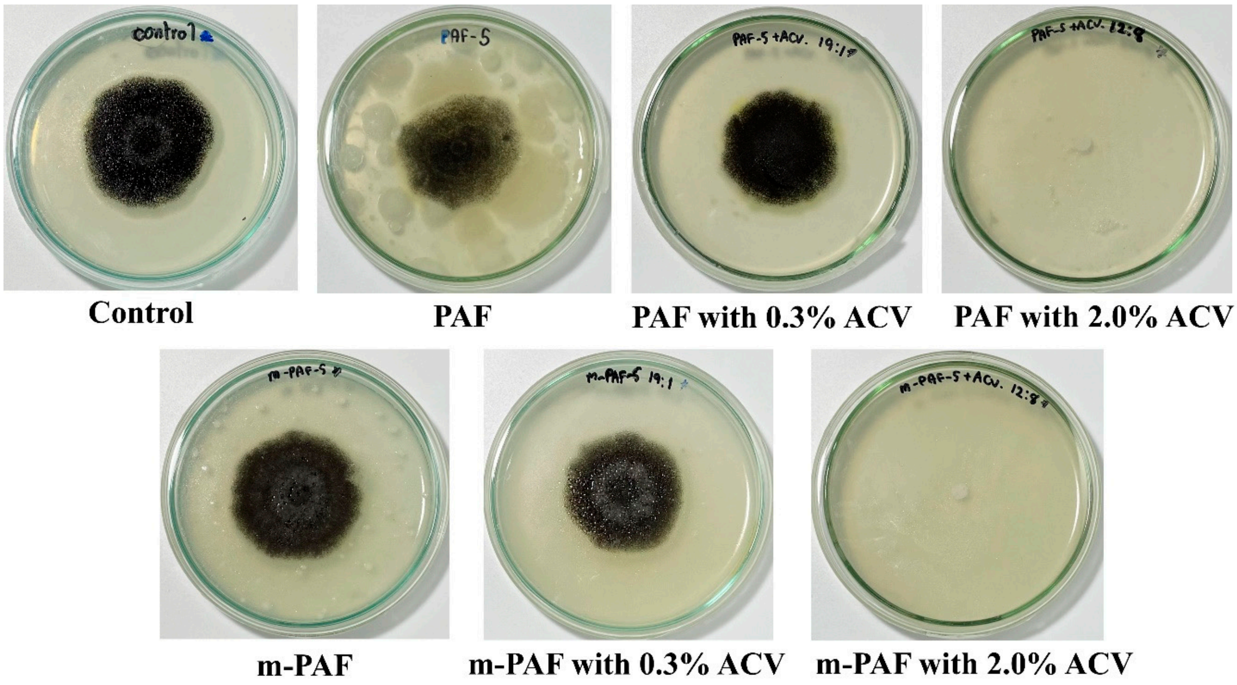

Figure S1. Microorganism growth monitored over a 7-day period in the adhesive systems. (a) control, (b) PAF water glue, (c) PAF with 0.3% ACV water glue, (d) PAF with 2% ACV water glue, (e) m-PAF water glue, (f) m-PAF with 0.3% ACV water glue, and (g) m-PAF with 2% ACV water glue. In unmodified starch (PAF) and starch ester (m-PAF) adhesives, ACV was employed at varying concentration as an antifungal agent against the *A. niger* fungus. The mycelial growth of *A. niger* fungus can be seen as dark shade in the plate, and white spots in PAF and m-PAF sample are the evidence of some bacterial growth. When 0.3% ACV is combined with the adhesives, white spots disappear.
